# Differential miRNA expression in inherently high- and low-active inbred mice

**DOI:** 10.14814/phy2.12469

**Published:** 2015-07-29

**Authors:** Michelle Dawes, Kelli J Kochan, Penny K Riggs, J Timothy Lightfoot

**Affiliations:** 1 Department of Health and Kinesiology, Texas A&M UniversityCollege Station, Texas, USA; 2 Department of Animal Science, Texas A&M UniversityCollege Station, Texas, USA

**Keywords:** miRNA, physical activity, wheel running

## Abstract

Despite established health benefits of regular exercise, the majority of Americans do not meet the recommended levels of physical activity. While it is known that voluntary activity levels are largely heritable, the genetic mechanisms that regulate activity are not well understood. MicroRNAs (miRNAs) are small non-coding RNAs that inhibit transcription by binding to a target gene, inhibiting protein production. The purpose of this study was to investigate differential miRNA expression between inherently high- (C57L/J) and low- (C3H/HeJ) active inbred mice in soleus, extensor digitorum longus (EDL), and nucleus accumbens tissues. Expression was initially determined by miRNA microarray analysis, and selected miRNAs were validated by qRT-PCR. Expression of 13 miRNAs varied between strains in the nucleus accumbens, 20 in soleus, and eight in EDL, by microarray analysis. Two miRNAs were validated by qRT-PCR in the nucleus accumbens; miR-466 was downregulated (∼4 fold; *P* < 0.0004), and miR-342-5p was upregulated (∼115 fold; *P* < 0.0001) in high-active mice. MiR-466 was downregulated (∼5 fold; *P* < 0.0001) in the soleus of high-active mice as well. Interestingly, miR-466 is one of several miRNA families with sequence located in intron 10 of *Sfmbt2*; miRNAs at this locus are thought to drive imprinting of this gene. “Pathways in cancer” and “TGF*β* signaling” were the most significant pathways of putative target genes in both the soleus and nucleus accumbens. Our results are the first to consider differential miRNA expression between high- and low-active mice, and suggest that miRNAs may play a role in regulation of physical activity.

## Introduction

There is a strong genetic component to the regulation of daily activity, with common environment accounting for only 3–6% of the variation (for review see Lightfoot 2011). Several potential candidate genes with regulatory roles in voluntary activity have been proposed, although none have shown a dramatic impact on phenotype (Lightfoot 2011). Thus, it is more likely that voluntary activity is regulated by many genes and/or many biological mechanisms, each with small effects. Our past work has led to speculation that intragenic, non-coding regions of the genome may function in regulation of the coding genes involved in innate physical activity levels (Lightfoot et al. 2010). Therefore, regulatory mechanisms such as microRNAs (miRNAs) that arise from these intragenic areas, are of interest in relation to complex phenotypes, and to date, have not been examined in relationship to voluntary physical activity levels.

MiRNAs are short sequences (∼22 nucleotides) of non-coding RNA that regulate gene expression post-transcriptionally by binding to a target mRNA sequence. This binding creates a physical block, preventing translation machinery from progressing down an mRNA strand, or by degrading the mRNA strand and preventing protein production altogether. Inside the nucleus, the “pri-miRNA” is transcribed from a sequence within intronic regions of DNA, which is cleaved and exported to cytoplasm as a single-loop structure referred to as the pre-miRNA (mir; (Cullen 2004)). Finally, the mature miRNA (miR) is single-stranded and produces a primary and secondary (denoted with *) sequence (Ambros et al. 2003). Strongly associated miRNA sequences are designated with suffixed letters, for example, miR-466b and miR-466d, which while quite similar, are derived from separate precursor sequences (Ambros et al. 2003).

MiRNAs have been shown to be responsive to exercise training, in both humans and mice, mostly in skeletal muscle (termed “myomirs”) and to a limited extent, blood plasma. Endurance training (Aoi et al. 2010; Baggish et al. 2011; Keller et al. 2011; Bye et al. 2013; Tonevitsky et al. 2013; Uhlemann et al. 2014) affects expression of different miRNAs than those identified in resistance exercise protocols (McCarthy and Esser 2007; Drummond et al. 2008; Davidsen et al. 2011; Sawada et al. 2013). Additionally, differential expression of some miRNAs occur immediately post-acute exercise bout, while other changes are not observed until weeks into training. Thus, alteration of miRNAs as a response to exercise stimulus is becoming well established. However, there has been much less work performed to suggest that miRNAs regulate the response to exercise. Davidsen et al. (Davidsen et al. 2011) evaluated miRNAs that differed between high and low responders to a 12 week resistance training protocol and showed that expression of miRNAs 378, 29a, 26a was diminished in individuals who were low responders, but did not change in high responders, while expression of miR-451 increased only in low responders. These authors also observed a correlation with abundance of miR-378 and muscle mass. Therefore, endogenous miRNA levels may contribute to individual variation in exercise response leading us to hypothesize that inherent miRNA expression may contribute to the regulation of physical activity levels as well.

Exercise capability through skeletal muscle function is only a part of the voluntary activity puzzle. The nucleus accumbens is considered the central reward center in the brain and was determined by previous work in our laboratory to be a potential site of activity regulation (Knab et al. 2009a, 2012; Ferguson et al. 2013). Dreyer (Dreyer 2010) also suggested that miRNAs played a role in drug addiction and brain neuroplasticity, often affecting the mesolimbic dopaminergic pathway. Knab et al. (Knab et al. 2009a) showed that decreased dopaminergic activity in the nucleus accumbens (which is a part of the mesolimbic pathway) is associated with increased activity levels in high-active mice, accompanied by lower D1 receptor and tyrosine hydroxylase expression levels. Some of these midbrain specific miRNAs involved with addiction are also involved with neurodegenerative ailments including Parkinson’s disease. Kim et al. (Kim et al. 2007) established that a miRNA expressed in brain dopaminergic neurons was diminished in Parkinson’s patients. Therefore, miRNAs may affect the endogenous central command drive to be active as well.

The purpose of this study was to determine whether systemic miRNA expression differences between inherently high-active and low-active mice can be observed in the brain (nucleus accumbens) and peripheral musculoskeletal (soleus and EDL) tissues. Knowledge of miRNA variability between high- and low-active mice will provide insight into the genetic regulatory mechanisms potentially causing differing levels of activity between these strains.

## Methods

Our laboratory has repeatedly shown that C3H/HeJ inbred mice are low-active, with an average daily wheel running distance of 2.93 ± 0.57 km, and C57L/J inbred mice are high-active, running 7.95 ± 0.96 km on average daily (Lightfoot et al. 2004, 2008, 2010). Additionally, Knab, et al. (Knab et al. 2009b) showed voluntary wheel running within these strains is highly repeatable. In the current study, 12 (6♂, 6♀) C57L/J and 12 (6♂, 6♀) C3H/HeJ mice that were 8-weeks old (56 days; Jackson Labs, Bar Harbor, ME) were housed individually. Previous work from our laboratory has indicated that gene expression within strain can be altered by running-wheel exposure (Dawes et al. 2014), and thus mice in this study were housed with a “locked” running wheel (450 mm circumference, Ware Manufacturing, Phoenix, AZ) to provide the same enrichment environment without running activity and eliminating the potential for activity-induced changes in the miRNA profile. Food (Harland Tekland 8604 Rodent Diet, Madison, WI) and water were provided ad libitum. All mice were housed in the same room of the university vivarium with 12 h light/dark cycles and with temperature and humidity maintained at 19–21°C and 40–50%, respectively. After 1 week, the mice were anesthetized with 2–4% isoflurane and subsequently euthanized. The nucleus accumbens, soleus, and EDL muscle were harvested and flash frozen in liquid nitrogen, and then stored at −80°C for later analysis. Body composition was analyzed prior to sacrificing using the Lunar Piximus DEXA (dual-energy X-ray absorptiometry) instrument (Fitchberg, WI). All procedures were approved by the Texas A&M University Institutional Animal Care and Use Committee.

Total RNA was extracted from nucleus accumbens, soleus, and EDL using the Qiagen miRNeasy kit (Qiagen, Valencia, CA), with inclusion of the on-column DNA treatment, according to the manufacturer’s instructions. RNA was quantified on a Nanodrop 1000 spectrophotometer (Thermo Scientific, Waltham, MA) and quality of RNA was assessed with an Agilent 2100 Bioanalyzer (Santa Clara, CA). Samples were chosen for arrays based on RNA integrity number (RIN) number and evaluation of the electropherogram provided by the Bioanalyzer, with samples having a RIN quality value of >7.5. Four male high-active and four male low-active samples from each tissue were evaluated by miRNA microarray (24 arrays total), assigned to an array at random. Agilent mouse miRNA microarrays (miRbase 17.0) – one color arrays with cyanine 3-Cp labeling – were used to detect global differential expression of miRNAs between high- and low-active animals from each of the three tissues. One hundred nanograms of total RNA in 2 *μ*L was labeled and the entire mass of RNA was used in hybridization. Optional labeling and hybridization spike-ins were used, as suggested by the manufacturer, as well as the purification of labeled RNA on the Bio-Rad microbiospin columns (Hercules, CA). Additionally, glass array slides were immersed in Agilent’s washing and stabilization solution for protection against additional degradation by ozone. Arrays were immediately scanned with an Agilent G2505C Scanner at three micron resolution, using GEML file 035430_D_F_20121221 for feature extraction and the miRNA_107_Sept09 protocol. The extended range option was also used with the photo multiplier lower setting at 0.05.

Quality reports of individual arrays were evaluated; subsequently, some arrays were dismissed due to heterogeneity of probe expression across array. Data were analyzed via Agilent GeneSpring 12.5 GX software. Probes with expression less than background were considered “not detected” and were subsequently removed from the analysis in the nucleus accumbens and soleus files, and “edited” entity lists were manually imported into GeneSpring for further analysis. Statistical analyses were not affected by any “less than background” probes for the EDL. Retained arrays were separated by tissue and subsequently normalized by percentile shift and background noise of arrays was removed (“background” was considered as the bottom 20% signal intensity). A moderated t-test was used to identify differential miRNA expression between activity levels (as arrays were already separated by tissue) with the alpha value set a priori <0.05 and fold change (FC) ≥2.0. Benjamini-Hochberg false discovery rate (FDR) was also applied to account for multiple testing and the resulting statistical test was the “corrected *P*-value” which was defined as *P* < 0.05 (aka: *q*-value). All microarray information is MIAME (Minimum Information About a Microarray Experiment) compliant and has been deposited in NCBI’s Gene Expression Omnibus (Edgar et al. 2002), accessible through GEO Series accession number GSE56374 (http://www.ncbi.nlm.nih.gov/geo/query/acc.cgi?acc=GSE56374).

Putative target genes of differentially expressed miRNAs were determined after GeneSpring analysis, incorporating predictions from TargetScan, PITA, PicTar, and/or microRNA.org, with miRNA to gene matching considered significant at *P* < 0.10. Putative target genes were then analyzed using DAVID (Database for Annotation, Visualization and Integrated Discovery v6.7; [da Huang et al. 2009] to determine predicted pathways and gene ontology (GO) terms, providing some insight into miRNA function. Putative target genes of soleus and EDL were also compared to differentially expressed proteins in soleus and EDL from a similar (age, living and harvesting conditions, no wheel, same number of each sex) cohort of C57L/J and C3H/HeJ mice from our laboratory, determined using two dimensional gel electrophoresis and subsequent mass spectrometry (Ferguson et al. 2014). MiRNAs warranting confirmation were selected by targets having similarity to differentially expressed proteins, and/or having a high fold change (FC) between strains, and/or GO terms with functional relevance to physical activity.

Validation of candidate miRNAs was carried out using Taqman® miRNA qRT-PCR assays (Life Technologies, Grand Island, NY). All miRNAs examined are listed in Table1. A fivefold RNA dilution series was performed to determine optimal quantity of input RNA into the reverse transcription reaction, for each assay in each tissue. RNA from three samples including both sexes and strains were combined to make a heterogeneous pool of RNA for the dilution series beginning with 200 ng total RNA. TaqMan® microRNA reverse transcription kit (cat. No. 4366596) and the miRNA specific RT primer were used for reverse transcribing the miRNA. RNA (200 ng, as determined from the standard curve) was input into all RT reactions, contained in 15 *μ*l, and cycled under the following conditions: 16°C for 30 min, 42°C for 30 min, and 85°C for 5 min. The PCR product was diluted 1:2 in 25 ng/*μ*L tRNA (Invitrogen, Carlsbad, CA) prior to qRT-PCR assays. Two *μ*L cDNA was combined with 10.0 *μ*L Taqman® universal PCR master mix - AmpErase® UNG (cat. No. 4324018), 1.0 *μ*L miRNA specific primer, and 7.0 *μ*L nuclease-free water in each qRT-PCR reaction. U6 snRNA and miR-99b-5p were used as endogenous reference miRNAs. MiR-99b-5p was selected for use as a reference as it was not differentially expressed on microarrays between activity levels (*P* = 0.94). The human analog of this assay, hsa-miR-99b-5p, was used (Table1). Additionally, the miR-466p-3p assay was used in place of the miR-466b-3p assay, as it was the most homologous assay available at the time (Table1). BLAST search of miR-466b-3p and miR-466p-3p showed that the sequences have 100% identity matching. qRT-PCR cycles were run in triplicate on an Applied Biosystems 7900HT Fast Real-Time PCR System (Carlsbad, CA). Amplification data were analyzed with Sequence Detection Software v. 2.2.2 (Applied Biosystems). Relative quantification was determined using the ΔΔCt method (Livak and Schmittgen 2001). The average ΔCt for all samples was used as the “reference” sample.

**Table 1:** miRNA associations and qRT-PCR assay information

miRNA family	Tissue miRNA evaluated in	miRNA validated by qPCR	Name of assay validated	Taqman assay ID
Small nuclear RNA	All	U6	miRNA, U6 snRNA	001973
mir-99	All	miR-99b-5p	hsa-miR-99b-5p	000436
mir-466	NA	miR-466d-3p	mmu-miR-466d-3p	002535
	Soleus	miR-466b-3p	mmu-miR-466p-3p	464896 mat
mir-342	NA	miR-342-5p	mmu-miR-342-5p	002527
mir-1960	Soleus	miR-1960	mmu-miR-1960	121148 mat

Describes the miRNA family of origin, tissue in which each miRNA was evaluated by qRT-PCR, and the name and Taqman ID of specific assay validated. Human miRNA 99b-5p was utilized as a control miRNA. MiR-466p-3p assay was used as miR-466b-3p in the soleus. Finally, as miR-466d-3p and miR-466b-3p are 100% matching, they are noted under the same miRNA family and referred to as simply miR-466 regardless of tissue of reference.

To determine whether U6 or miR-99b-5p were the most appropriate endogenous control to use, a modification to the ΔΔCt calculation was used to create a ratio (2^ΔCt^ with ΔCt = sample Ct − “reference”). Differential expression was then determined using a chi-square test for each miRNA assay in each tissue, as well as controls, with alpha values set a priori at 0.05 (JMP™ Pro10.0, SAS Institute, Cary, NC). Further analysis of the U6 and miR-99b-5p included calculation the coefficient of variation (CoV) for both potential endogenous controls within tissue. In all analyses, Ct values that were greater than 2.5 standard deviations away from the mean were considered outliers and eliminated from the dataset. If differential expression was observed, data were subsequently analyzed for sex differences.

## Results

No difference in percent body fat (*P* < 0.107; 14.2 g ± 1.8 g C3H/HeJ; 12.8 g ± 2.0 g C57L/J; mean ± SD) or weight (*P* < 0.178; 20 g ± 3.3 g C3H/HeJ; 21.7 g ± 3.7 g C57L/J; mean ± SD) was observed between the strains. The five microarrays removed due to poor signal quality included two low-active soleus, two high-active EDL, and one low-active nucleus accumbens. Hsa-miR-99b-5p proved an adequate control assay for all three tissues, not differing in expression between strains within tissues (*P* = 0.69 for nucleus accumbens, *P* = 0.644 for EDL, and *P* = 0.225 for soleus). Although U6 was shown to be similar between strains in the soleus (*P* = 0.09) and EDL (*P* = 0.13), it was different between strains in the nucleus accumbens (*P* = 0.02). Table2 shows that in comparison to U6, miR-99b-5p had a smaller CoV overall, providing additional merit for use of miR-99b-5p as our endogenous reference miRNA. Therefore, all expression results are presented using miR-99b-5p as the reference miRNA.

**Table 2:** Variability of U6 and miR-99b as reference assays

Variability of U6 and miR-99b as reference assays				
	hsa-miR-99b		U6	
	*P*-value	C of V	*P*-value	C of V
Nucleus accumbens	0.69	0.40	0.024*	0.71
EDL	0.64	0.22	0.13	0.86
Soleus	0.23	0.23	0.09	0.21

The *P*-value and coefficient of variation (CoV) for each tissue using miR-99b and U6 as endogenous reference miRNAs. Hsa-miR-99b-5p proved an adequate control assay for all three tissues, not differing in expression between strains within tissues. Although U6 was shown to be similar between strains in the soleus, it was different between strains in the nucleus accumbens. The CoV was greater, in general, when using U6 as the reference miRNA, providing additional support for the use of miR-99b as our endogenous reference miRNA.

* Significantly different expression, *P* < 0.05.

**Figure 1:**
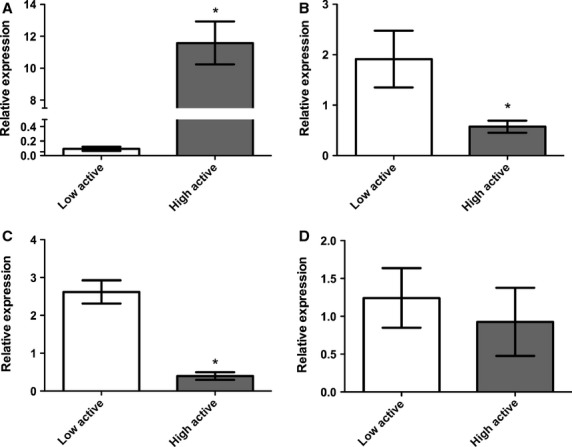
Relative expression determined by qRT-PCR of (A) miR-342-5p (*P* < 0.0001) and (B) miR-466 (*P* < 0.0004) in nucleus accumbens and (C) miR-466 (*P* < 0.0001) and (D) miR-1960 (*P* = 0.06) in the soleus between high- (C57L/J) and low- (C3H/HeJ) active mice. *denotes differential expression. Relative expression is mean ± standard deviation.

Thirteen miRNAs were differentially expressed between strains (*P* < 0.05) by microarray analysis in the nucleus accumbens (Table3). Sequences for seven of these 13 miRNAs are located in the proximal end of chromosome 2, specifically in intron 10 of the *Sfmbt2* gene, and all exhibited reduced expression in the high-active versus low-active mice. Of the 13 differentially expressed miRNAs, miR-342-5p (*P* < 0.0001; Fig.1A) and miR-466d-3p (*P* = 0.0004; Fig.1B) were chosen for validation by qRT-PCR. Additional analysis by sex indicated no difference between males and females for either miRNA (*P* = 0.603 and *P* = 0.134, respectively).

**Table 3:** Differentially expressed miRNAs between high- and low-active mice

Systematic name	*P* (Corr)^1^	Regulation (high vs low)	Fold change	Active sequence^2^	Chr‡	Mirbase accession No	Positon on chr	Strand	Stop position on chr
Nucleus Accumbens									
mmu-miR-3095-3p	<0.001	Up	104	AAAAGCTCTCTCTCCAGT	Chr4	MIMAT0014912	58453959	+	58453943
mmu-miR-342-3p	<0.001	Up	4	ACGGGTGCGATTTCTGT	Chr12	MIMAT0000590	109896897	−	109896912
**mmu-miR-342-5p**	<0.001	**Up**	**257**	CTCAATCACAGATAGCACC	Chrl2	MIMAT0004653	109896852	−	109896869
mmu-miR-375	0.03	Down	4	TCACGCGAGCCGAACG	Chr1	MIMAT0000739	74947292	+	74947278
mmu-miR-376c	<0.01	Up	3	ACGTGAAATTTCCTCTATGTT	Chrl2	MIMAT0003183	110960981	−	110961000
**mmu-miR-466d-3p**	<0.001	**Down**	**54**	CTATGTGTGCGTGTAT	Chr2	MTMAT0004931	10433650	−	10433664
mmu-miR-467a*	<0.01	Down	4	TGTAGGTGTGTGTATGTATA	Chr2	MTMAT0002108	10398019	−	10398038
mmu-miR-467b	<0.01	Down	3	CATATACATGCAGGCACT	Chr2	MIMAT0005448	10402887	−	10402903
mmu-miR-467 c	<0.001	Down	87	CACATATACATGCACGCAC	Chr2	MTMAT0004885	10395572	−	10395589
mmu-miR-5117	<0.001	Up	191	TAACTTTATTGATCATCACTAAC	Chr1	MIMAT0020625	162967492	−	162967513
mmu-miR-669f-3p	<0.01	Down	2	ATACGTGTGTGTGTATGT	Chr2	MTMAT0005839	10388917	−	10388934
mmu-miR-6691	<0.001	Down	194	ACATATACATGCACACAC	Chr2	MIMAT0009418	10390015	−	10390032
mmu-miR-669n	<0.01	Down	3	ACACACATCCACACACAA	Chr2	MIMAT0009427	10430950	−	10430967
EDL									
mmu-miR-1957	<0.001	Down	237	GTCATATGCTCTACCACT	Chr4	MTMAT0009430	118802399	−	118802415
mmu-miR-1966	<0.001	Down	80	GACTCTCTCCTGAGCC	Chr8	MTMAT0009439	108139391	−	108139405
mmu-miR-1967	<0.001	Down	68	GCATCTTCTCCCCAG	Chr8	MTMAT0009440	126546597	−	126546610
mmu-miR-205	<0.001	Down	326	CAGACTCCGGTGGAAT	Chr1	MIMAT0000238	195333684	+	195333670
mmu-miR-3091-5p	<0.001	Up	50	GCGGGCCCAACC	Chr2	MIMAT0014903	179992262	−	179992272
mmu-miR-342-3p	0.02	Up	5	ACGGGTGCGATTTCTGT	Chrl2	MIMAT0000590	109896897	−	109896912
mmu-miR-5117	<0.001	Up	120	TAACTTTATTGATCATCACTAAC	Chr1	MTMAT0020625	162967492	−	162967513
mmu-miR-5118	<0.001	Down	54	ACCAGGCTGGCCTA	Chrl6	MIMAT0020626	55494889	−	55494901
Soleus									
mmu-miR-148a	0.02	Down	2	ACAAAGTTCTGTAGTGCACT	Chr6	MIMAT0000516	51219892	+	51219874
mmu-miR-1843-5p	<0.001	Down	52	AGTCAGACAGAGACCTC	Chr12	MIMAT0014805	81492623	+	81492608
mmu-miR-1927	<0.001	Down	220	TCAGTCCCTAACATCCA	Chr1	MIMAT0009390	162226082	+	162226067
mmu-miR-193*	<0.001	Down	80	TCATCTTGCCCGCA	Chr11	MIMAT0004544	79525486	−	79525498
**mmu-miR-1960**	**<0.001**	**Down**	**101**	AGCCCTCTTCTAACAGC	Chr5	MIMAT0009433	30497297	−	30497312
mmu-miR-299	<0.001	Down	22	AAGCGGTTTACCGTCCC	Chrl2	MTMAT0004577	110948892	−	110948907
mmu-miR-3 26	<0.001	Down	42	ACTGGAGGAAGGGCCCA	Chr7	MIMAT0000559	106700843	−	106700858
mmu-miR-342-3p	<0.001	Up	5	ACGGGTGCGATTTCTGT	Chrl2	MIMAT0000590	109896897	−	109896912
mmu-miR-3 62-5p	<0.001	Down	31	ATTCACACCTAGGTTCCA	ChrX	MTMAT0000706	6819135	+	6819119
mmu-miR-376a	<0.001	Down	79	ACGTGGATTTTCCTCTA	Chrl2	MIMAT0000740	110962039	−	110962054
mmu-miR-431	<0.001	Down	42	TGCATGACGGCCTGC	Chrl2	MIMAT0001418	110828676	−	110828689
**mmu-miR-466b-3p**	**<0.001**	**Down**	**71**	TCTTATGTGTGCGTGTA	Chr2	MIMAT0004876	10395901	−	10395917
mmu-miR-467 a*	<0.001	Down	169	TGTAGGTGTGTGTATGTATA	Chr2	MIMAT0002108	10398019	−	10398038
mmu-miR-467b	<0.001	Down	227	CATATACATGCAGGCACT	Chr2	MIMAT0005448	10402887	−	10402903
mmu-miR-467e	<0.001	Down	73	ACATATACATGCTCACACT	Chr2	MIMAT0005293	10427362	−	10427379
mmu-miR- 5103	<0.001	Down	59	CCTCAGGGGATCCC	Chr1	MIMAT0020610	34490035	+	34490023
mmu-miR- 5117	<0.001	Up	204	TAACTTTATTGATCATCACTAAC	Chr1	MIMAT0020625	162967492	−	162967513
mmu-miR-582-5p	<0.001	Down	69	AGTAACTGGTTGAACAACTGTA	Chrl3	MIMAT0005291	110114949	−	110114969
mmu-miR-711	<0.001	Down	44	CTTACATCTCTCCCCG	Chr9	MIMAT0003501	108872022	−	108872036
mmu-miR-99a*	<0.001	Down	40	AGACCCATAGAAACGAGC	Chrl6	MIMAT0016981	77599226	−	77599242

Differentially expressed miRNAs (*P* < 0.05) between high- (C57L/J) and low-active (C3H/HeJ) mice strains in nucleus accumbens, EDL, and soleus.

1 “*P*(corr)” refers to adjusted *P* -value after multiple testing correction; fold change is adjusted for multiple testing correction.

2 “Active sequence” is the probe sequence, “strand” refers to sense (+) or anti-sense (−) strand on which miRNA sequence is located. “Chr” refers to the chromosome on which the miRNA is located. Bold miRNAs were selected for validation by qRT-PCR. Highlighted miRNAs in nucleus accumbens and soleus are located in proximal end of Chr. 2.

* Housekeeping miRNA.

‡ Chromosome number of miRNA.

In the soleus, 20 miRNAs were found to be differentially expressed between strains by microarray. Four of the miRNA sequences differentially expressed in the soleus were also located in intron 10 of the *Sfmbt2* gene, and were downregulated in the high-active animals, similar to the nucleus accumbens. MiR-466b-3p was amongst these four miRNAs (Table3), and was determined to be differentially expressed between high- and low-active animals by qRT-PCR analysis (*P* < 0.0001; Fig.1C), with no difference in expression between sexes (*P* = 0.908). In contrast to the microarray data, qRT-PCR analysis showed that miR-1960 only trended towards differential expression between strains (*P* = 0.060; Fig.1D).

MiR-466d-3p (differentially expressed in the nucleus accumbens) and miR-466b-3p (differentially expressed in the soleus) are a part of the same family of miRNAs (Table1). The mature miRNA sequences (∼21mer) for these miRNAs are 100% identical. However, the stem-loop sequence (∼80mer) is slightly different between these miRNAs (∼90% identical according to BLAST comparison), which differentiates them. As the mature sequences of these miRNAs were assayed by qRT-PCR, we will simply refer to them as miR-466. There are multiple probes on the microarray with 16 ± 2 nucleotides for these miRNAs (the probe used to determine differential expression is listed under “active sequence” on Table3), which provides distinction between these family members.

Finally, in the EDL, eight miRNAs were differentially expressed between strains as determined by microarray analysis (Table3). However, no further validation of these miRNAs was conducted at this time, as quantities of RNA (200 ng determined by dilution series) were insufficient for additional qRT-PCR assays, beyond establishment of endogenous controls.

KEGG (Kyoto Encyclopedia of Genes and Genomes) pathways of putative target genes generated through DAVID were observed for association with drive or capability to be physically active. Approximately 30.3% of putative target genes (*n* = 130) in the soleus were classified into 11 KEGG pathways (*P* < 0.01; Table4), 27.7% of putative target genes (*n* = 359) in NA were classified into 39 pathways (*P* < 0.01; Table5), and in the EDL, 31% of putative target genes (*n* = 173) were classified into 5 pathways (*P* < 0.01; Table6). The most significant predicted pathways for both soleus and NA included “pathways in cancer” and “TGF*β* signaling”. Additional pathways related to exercise capability that are noteworthy in the soleus are involved with vasculature development and muscle development (Table4). In the NA, the most significant GO terms involved transcription regulation, as well as terms relating to metabolism and organ development (Table5). Many GO terms in the EDL were identified pertaining to neuron development and nerve impulse, as well as dopamine secretion (Table6).

**Table 4:** KEGG pathways and GO terms for soleus putative target genes

	No. of genes included	% of genes included	*P*-value Benjamini adjusted
KEGG pathways for putative target genes - soleus			
Pathways in cancer	19	4.4	3.50E-02
TGF-beta signaling pathway	9	2.1	4.20E-02
Cell cycle	10	2.3	8.70E-02
Renal cell carcinoma	7	1.6	1.30E-01
Ubiquitin mediated proteolysis	8	1.9	5.60E-01
Progesterone-mediated oocyte maturation	6	1.4	5.80E-01
Cytokine-cytokine receptor interaction	11	2.6	5.70E-01
Toll-like receptor signaling pathway	6	1.4	6.80E-01
Endocytosis	9	2.1	7.00E-01
Adherens junction	5	1.2	6.90E-01
Type II diabetes mellitus	4	0.9	6.80E-01
GO terms for putative target genes - soleus			
Vasculature development	14	3.3	1.90E-01
Blood vessel development	13	3	2.70E-01
Blood vessel morphogenesis	11	2.6	3.30E-01
Regulation of cellular response to stress	6	1.4	4.10E-01
Angiogenesis	8	1.9	4.60E-01
Hemopoiesis	12	2.8	4.60E-01
Muscle tissue development	8	1.9	4.80E-01
Regulation of vasoconstriction	3	0.7	4.80E-01
Muscle organ development	9	2.1	5.40E-01
Striated muscle tissue development	7	1.6	6.10E-01

Predicted pathways and gene ontology (GO) terms of differentially expressed miRNA target genes from soleus analyzed through DAVID. No. of genes and percent of genes; number of genes out of all putative target genes generated through Genespring software included in respective KEGG pathway or GO term category.

**Table 5:** KEGG pathways and GO terms for nucleus accumbens putative target genes

	No. of genes included	% of genes included	*P*-value Benjamini adjusted
KEGG pathways for putative target genes - nucleus accumbens			
Pathways in cancer	48	3.7	3.50E-06
TGF-beta signaling pathway	21	1.6	1.90E-05
Endocytosis	31	2.4	3.30E-04
Cell cycle	21	1.6	4.40E-03
MAPK signaling pathway	31	2.4	3.00E-02
Hypertrophic cardiomyopathy (HCM)	14	1.1	4.90E-02
Colorectal cancer	14	1.1	5.20E-02
Adipocytokine signaling pathway	12	0.9	5.00E-02
Ubiquitin mediated proteolysis	18	1.4	7.50E-02
Cytokine-cytokine receptor interaction	27	2.1	7.30E-02
Cell adhesion molecules (CAMs)	19	1.5	9.70E-02
Prostate cancer	13	1	1.20E-01
Renal cell carcinoma	11	0.8	1.30E-01
Focal adhesion	22	1.7	1.20E-01
Dilated cardiomyopathy	13	1	1.10E-01
Insulin signaling pathway	17	1.3	1.10E-01
Bladder cancer	8	0.6	1.20E-01
Hematopoietic cell lineage	12	0.9	1.20E-01
Arrhythmogenic right ventricular cardiomyopathy (ARVC)	11	0.8	1.40E-01
Chronic myeloid leukemia	11	0.8	1.40E-01
Adherens junction	11	0.8	1.40E-01
Wnt signaling pathway	17	1.3	1.60E-01
Heparan sulfate biosynthesis	6	0.5	1.60E-01
Acute myeloid leukemia	9	0.7	1.50E-01
Type II diabetes mellitus	8	0.6	1.90E-01
Melanoma	10	0.8	1.80E-01
Thyroid cancer	6	0.5	1.80E-01
ECM-receptor interaction	11	0.8	1.80E-01
ErbB signaling pathway	11	0.8	2.20E-01
Glioma	9	0.7	2.20E-01
mTOR signaling pathway	8	0.6	2.30E-01
Chemokine signaling pathway	18	1.4	2.70E-01
Axon guidance	14	1.1	2.80E-01
Long-term potentiation	9	0.7	2.90E-01
Pancreatic cancer	9	0.7	3.20E-01
SNARE interactions in vesicular transport	6	0.5	3.30E-01
Gap junction	10	0.8	3.30E-01
NOD-like receptor signaling pathway	8	0.6	3.30E-01
Melanogenesis	11	0.8	3.30E-01
GO terms for putative target genes - nucleus accumbens			
Regulation of gene expression	276	21.3	1.80E-12
Regulation of transcription	252	19.5	2.10E-11
Regulation of transcription, DNA-dependent	170	13.1	1.40E-07
Positive regulation of gene expression	72	5.6	2.90E-06
Positive regulation of transcription	70	5.4	4.60E-06
Organ morphogenesis	80	6.2	1.60E-05
Embryonic organ development	40	3.1	1.90E-04
Tissue development	78	6	7.00E-04
Positive regulation of cell differentiation	29	2.2	4.80E-03
Heart growth	3	0.2	6.50E-01

Predicted pathways and gene ontology (GO) terms of differentially expressed miRNA target genes from NA analyzed through DAVID. No. of genes and percent of genes; number of genes out of all putative target genes generated through Genespring software included in respective KEGG pathway or GO term category.

**Table 6:** KEGG pathways and GO terms for EDL putative target genes

	No. of genes included	% of genes included	*P*-value Benjamini adjusted
KEGG pathways for putative target genes - EDL			
Oocyte meiosis	9	1.6	9.50E-01
Pathways in cancer	17	3.1	9.00E-01
GnRH signaling pathway	7	1.3	9.70E-01
Basal transcription factors	4	0.7	9.30E-01
Prion diseases	4	0.7	9.20E-01
GO terms for putative target genes - EDL			
Tissue development	33	5.9	6.00E-01
Regulation of transmission of nerve impulse	10	1.8	7.10E-01
Protein targeting	11	2	7.50E-01
Regulation of neurological system process	10	1.8	7.00E-01
Regulation of programmed cell death	28	5	7.50E-01
Neurogenesis	27	4.9	7.40E-01
Regulation of synaptic transmission	9	1.6	7.00E-01
Neuromuscular synaptic transmission	4	0.7	7.10E-01
Spinal cord motor neuron differentiation	4	0.7	7.60E-01
Generation of neurons	24	4.3	7.60E-01
Tissue morphogenesis	14	2.5	7.60E-01
Central nervous system development	19	3.4	7.60E-01
Neuron development	16	2.9	7.50E-01
Neuron projection development	13	2.3	7.40E-01
Epithelial cell differentiation	9	1.6	7.30E-01
Spinal cord development	5	0.9	7.80E-01
Hemopoietic progenitor cell differentiation	3	0.5	7.70E-01
Water transport	3	0.5	7.70E-01
Cellular protein metabolic process	75	13.5	7.90E-01
Axonogenesis	10	1.8	7.90E-01
Ion homeostasis	15	2.7	7.80E-01
Neuron migration	6	1.1	7.90E-01
Positive regulation of dopamine secretion	2	0.4	7.80E-01
Hemopoiesis	13	2.3	8.00E-01
Neuron projection morphogenesis	10	1.8	8.20E-01
Synaptic transmission	10	1.8	8.30E-01
Positive regulation of actin filament depolymerization	2	0.4	8.30E-01
Positive regulation of integrin activation	2	0.4	8.30E-01
Positive regulation of catecholamine secretion	2	0.4	8.30E-01
Unsaturated fatty acid metabolic process	4	0.7	8.40E-01
Cell morphogenesis involved in neuron differentiation	10	1.8	8.40E-01

Predicted pathways and gene ontology (GO) terms of differentially expressed miRNA target genes in EDL analyzed through DAVID. No. of genes and percent of genes; number of genes out of all putative target genes generated through Genespring software included in respective KEGG pathway or GO term category.

## Discussion

It has been well established that a genetic component to voluntary physical activity levels exists and is independent of environment. However, voluntary physical activity is a complex phenotype, with evidence of regulation from both central and peripheral mechanisms (e.g. (Ferguson et al. 2014; Kelly et al. 2012)), making elucidation of the responsible mechanisms difficult. This project has shown that differential miRNA expression in the nucleus accumbens, soleus, and EDL is associated with differing inherent levels of physical activity. Specifically, miRNA microarray analysis identified 13 miRNAs in nucleus accumbens, eight in EDL, and 20 in soleus to be differentially expressed between high- and low-active strains of mice. Furthermore, miR-342-5p and miR-466 in the nucleus accumbens, and miR-466 in soleus were validated by qRT-PCR to be differentially expressed between the high- and low-active mice. These differential miRNA expression patterns may be contributors to regulation of physical activity.

Interpretation of the mechanisms regulating voluntary physical activity began with genomic analysis for QTL associated with physical activity in both human and animal populations, from which several candidate genes were proposed (Lightfoot 2011). However, as we have noted recently (Dawes et al. 2014), and as was eloquently observed by Flint, et al. (Flint et al. 2005), deriving genes/proteins of interest from QTL can be difficult and infrequently successful. Therefore, it was not surprising when a comparison of the genomic position of all differentially expressed miRNAs (Table3) to previously identified activity QTL in these strains (Lightfoot et al. 2008, 2010; Leamy et al. 2011) indicated no overlap in location between the miRNA sequence and QTL. While correspondence of differentially expressed miRNA sequence to a QTL would be interesting, genome association studies utilize genomic DNA, in which sequence variability does not necessarily correspond to mRNA expression levels (Dawes et al. 2014). Recently, however, Ferguson and colleagues from our laboratory (Ferguson et al. 2014), have taken a “protein to gene” approach to uncovering regulation of voluntary activity by determining the proteome signature in peripheral tissue (EDL and soleus) of inherently high- and low-active mice, in attempts to better understand systemic variability between activity levels. The present miRNA study is an “intermediate” investigation of regulatory mechanisms, evaluating potential causative factors for the variable proteome observed by Ferguson, et al. (Ferguson et al. 2014).

In this study, we observed miR-466 in the nucleus accumbens and soleus to be differentially expressed between the strains. Specifically, miR-446d-3p in the nucleus accumbens and miR-466b-3p in the soleus, were downregulated in the high-active mice (Table3; Fig.1B and C). The mature sequences of these miRNAs are matching, although different qRT-PCR assays were utilized for validation (see Table1). Both primary sequences are located in intron 10 of *Sfmbt2* (“Scm-like with four mbt domains 2” gene) on the proximal end of mouse chromosome 2. *Sfmbt2* has a similar genetic structure to the polycomb-group proteins, which are capable of chromatin remodeling and are known for silencing HOX genes (Lee et al. 2013). In mice and rats only, *Sfmbt2* is imprinted in early embryos and extra-embryonic tissues, and contains sequences for many small RNAs (Kuzmin et al. 2008; Wang et al. 2011). It is suggested that it is the miRNA cluster that is causative of the imprinting at this locus and that most (∼84%) of imprinted gene/miRNA arrangements are conserved in humans (Wang et al. 2011). This circumstance seems an uncommon genomic event; Wang, et al. (Wang et al. 2011) states that *Sfmbt2* has been “caught in the act of becoming imprinted”. Therefore, as syntany is not conserved between human and mouse in this region, structure and regulation of this gene between human and mouse may also differ. Although these results bring to light limitations in translating miRNA results from mouse model to human, the magnitude of differential expression of these miRNAs between strains and their ability to imprint genes, gives merit to epigenetic modifications that may be key drivers in activity levels, especially in rodent models.

Several other miRNAs in the nucleus accumbens and soleus that we observed to be differentially expressed by microarray are also located in intron 10 of *Sfmbt2* (see Table3). MiR-669 is a part of this miR-297-669 cluster of miRNAs, shown by Druz, et al. (Druz et al. 2012) to be upregulated under nutrient depleted conditions in cell culture. Specifically, these authors showed that glucose deprivation caused oxidative stress, leading to histone acetylation of the miRNA promoter region, and activating expression of miR-466 h-5p and *Sfmbt2*. Therefore, considering expression of this miRNA cluster was diminished in our high-active animals compared to low active, we speculate that endogenous basal metabolic and ROS production differences may play a significant role in regulating voluntary activity levels. Thus, further studies should be conducted to validate similar functionality of miRNAs in this cluster, as well as expression level of *Sfmbt2* between the high- and low-active strains.

Expression of miR-466 in the soleus was ∼5 fold lower in high-active mice. Target gene analysis of miR-466 showed some similarity in gene families to Ferguson, et al.’s previous protein work (Ferguson et al. 2014). For example, phosphofructokinase, liver, type B (*Pfkl* – *P* = 0.04) was a target gene of miR-466b-3p, and 6-phosphofructo-2-kinase/fructose-2,6-biphosphatase 1 (*Pfkfb1*) protein was shown to be expressed higher in the low-active mice (Ferguson et al. 2014). The *Pfkfb1* gene produces a class of “bifunctional” enzymes that maintain levels of fructose-2,6-bisphosphatase (F-2,6-BP), which is required for glycolysis, and is specific to liver, heart, and skeletal muscle (Minchenko et al. 2003). The kinase portion of the enzyme synthesizes F-2,6-BP and the phosphatase degrades F-2,6-BP (Okar et al. 2001). Fructose-2,6-BP is the most powerful activator of the *Pfk* enzymes (Pilkis et al. 1995), which regulate glucose through glycolytic pathways. It is intriguing to note that the regulatory pathway of two important metabolic enzymes were differentially expressed between high- and low-active mice in this miRNA study and the previous proteomic study (Ferguson et al. 2014), indicating that inherent differences in basal metabolism in skeletal muscle may have a regulatory role on voluntary physical activity levels.

In the nucleus accumbens, miR-342-5p was found to be upregulated (∼115 fold) and miR-466 was downregulated (∼4 fold) in the high-active mice. Target gene prediction found *Tceanc* (transcription elongation factor A (SII) N-terminal and central domain containing on the X chromosome) to be a target gene for both of these miRNAs (*P* = 0.03). While opposite trends in expression patterns exists between these two miRNAs, remembering that miRNAs bind to a target gene to elicit action, the greater relative fold change of miR-342-5p may be producing diminished protein expression of *Tceanc*, as well as other genes, in the high-active animals. Recent work by Ferguson, et al. (Ferguson et al. 2015) suggests that protein expression of transcription elongation factor A 3 (*Tcea3*) is differentially expressed in the nucleus accumbens between high- and low-active mice. We find the gene function similarities noteworthy as indicators of the types of genes that are potentially systemic regulators of inherent activity in the nucleus accumbens, although more work needs to be carried out to investigate the interaction between these miRNA and protein levels.

Both soleus and nucleus accumbens putative target genes are included in KEGG pathways “pathways in cancer” and “*Tgfβ* signaling”. “Pathways in cancer” included genes such as *Wnt9a, Shh*, and zinc finger transcription factors, suggesting involvement in cell cycle. *Tgfβ* signaling (also including *Tnf* and *Bmp5*) is known to be involved in exercise, limiting skeletal muscle myogenesis (Coffey and Hawley 2007). *Tnf* and *Shh* were also identified in the muscle tissue development, angiogenesis, and vasculature development GO term categories. Consequently, miRNAs identified as differentially expressed regulate these pathways, leading to the basis for physiological differences in inherent voluntary wheel running between these strains. Keller, et al. (Keller et al. 2011) observed upregulation of genes associated with cardiovascular and embryonic tissue development in men that were high responders to a 6 week cycling program. These authors identified TGF*β* as a top canonical pathway, as well as calcium through camodulin-dependent protein kinase (CamKII). *CamKII* is a putative target gene for miR-669f-3p, miR-342-3p, and miR-466d-3p in the nucleus accumbens. Similar to our findings, the aforementioned study determined that 22% of the miRNA gene targets differentially expressed between their high and low responders fell into GO category of transcription regulation. Specifically, Keller, et al. (Keller et al. 2011) notes that SOX9 was involved in muscle adaption to aerobic training; *SOX9* was a target for both miR-342-3p, and miR-466d-3p in this study as well. Wang, et al. (Wang et al. 2014) investigated differential miRNA expression between 2- and 4-cell developing C57BL/6 mouse embryos by miRNA microarray. Their results identified miR-466d-3p to be upregulated in 4-cell compared to 2-cell embryos. MiR-467b*, miR-669i, and miR-669 m-3p were also differentially expressed between developmental stages. These studies provide greater merit that our differentially expressed miRNAs may have regulatory roles on voluntary activity levels through developmental genes.

Finally, although no validation of miRNAs was performed in the EDL, the significance of the EDL is worth mentioning briefly. The EDL is a fast-twitch, oxidative muscle fiber muscle in the lower leg of rodents. While we presume that slow-twitch, oxidative muscle fibers are primarily engaged for long duration wheel running usually exhibited by the high-active animals, we do not disregard the potential involvement of fast-twitch fibers. Suwa, et al. (Suwa et al. 2003) observed rats that were inbred for fast-twitch fiber dominance actually had higher levels of voluntary wheel running than non-fast-twitch dominant controls. Our microarray results showed eight miRNAs to be differentially expressed between strains in the EDL; however, as previously stated, insufficient quantities of RNA were available for qRT-PCR assays beyond the endogenous controls and thus, validation of the EDL results awaits future studies.

In conclusion, this study presents the differential miRNA signature of inherently high- and low- active inbred mice. In the nucleus accumbens, miR-342-5p and miR-466 were validated as differentially expressed between strains by qRT-PCR, as was miR-466 in the soleus. Further experiments to determine the role of these miRNAs in activity regulation are needed including use of miRNA antagonists to down-regulate the differentially expressed miRNAs identified in this study, investigation of *Sfmbt2* expression levels, and analysis of the interaction between miRNAs and selected target genes. Regardless, these results demonstrate the importance of miRNA gene expression and contribute additional insight into the regulatory mechanism of voluntary physical activity.
